# Teaching Autonomic Cardiovascular Pharmacology to Medical Students: An Innovative Worksheet Approach

**DOI:** 10.15694/mep.2021.000065.1

**Published:** 2021-03-08

**Authors:** Christopher Cooley, Mary Jo Trout

**Affiliations:** 1 Wright State University Boonshoft School of Medicine

**Keywords:** Autonomic Pharmacology, ANS Drugs, Worksheet

## Abstract

This article was migrated. The article was marked as recommended.

**Introduction:** Autonomic (ANS) pharmacology is a fundamental part of any medical education curriculum and presents unique learning challenges. Students at a US allopathic medical school created a worksheet as a novel approach to improving student learning of and confidence with cardiovascular (CV) ANS pharmacology during the pre-clinical curriculum.

**Methods:** The student who created the cardiovascular ANS worksheet introduced it as a supplemental learning tool to the class of 2023 in their first semester of the first year in a foundational biomedical science course. The worksheet allows students to draw changes in heart rate and blood pressure when ANS drugs are administered individually and in combination. A self-assessment tool was also created to provide students immediate feedback on their learning following the completion of the worksheet. Voluntary surveys evaluated amount of usage, confidence with, and usefulness of the worksheet in learning the topic. Course exam results of students who used the tool were compared with all students in the previous year’s class (control).

**Results:** More than half the class participated in using the worksheet, self-assessments, and the surveys. Not only did self-confidence with the topic significantly increase but so did their performance on ANS-relevant exam questions when comparing those who used the worksheet with the previous year’s class.

**Conclusions:** An innovative worksheet with self-assessment tool for learning CV ANS pharmacology holds promise to enhance confidence with this topic as well as improve learning outcomes as measured by course exams.

## Introduction

### Autonomic Pharmacology

Autonomic (ANS) pharmacology is a challenging concept for medical students to learn that requires practice to successfully master. At Wright State University Boonshoft School of Medicine (BSOM), reasons for the difficulty in learning this topic include: (i) ANS is the first concept requiring students to apply pharmacology principles to individual drugs and to the whole system; (ii) students often have difficulty relating ANS pharmacology cellular mechanisms to the broader system, such as the cardiovascular system, and formulating an understanding beyond the simple drug-receptor relationship; and (iii) integrating physiology with a drug’s cellular mechanism requires practice to develop a working understanding, especially when two ANS drugs are administered sequentially.

### Integrating Significant Learning into a Curriculum

At Wright State University BSOM, autonomic pharmacology is incorporated in several phases throughout the Foundations curriculum in the interest of spaced learning, long-term potentiation, and improved retrieval. Significant learning curriculum design asserts that acquiring knowledge alone is insufficient for long-term retention and application. However, when the knowledge is matched with a patient experience, then significant learning can occur ( Hurtubise and Roman, 2014). Didactic methods such as worksheets, flash cards, and games are a vital tool that promote critical thinking skills, increases student engagement, boosts confidence and enjoyment, and allows for a deeper understanding of concepts which are instrumental throughout a physicians’ career ( Hedrick and Young, 2008; Shiroma, Massa and Alarcon, 2011; Beylefeld and Struwig, 2007; Howard, Collins and DiCarlo, 2002; O’Leary *et al.*, 2005). By promoting self-directed and active engagement, students will be better equipped for lifelong learning as physicians ( Hurtubise and Roman, 2014).

At BSOM, students are introduced to ANS pharmacology by the 11 ^th^ week of medical school in Origins (an introductory course to basic medical science). The Origins course lays the foundation in the basic cellular physiology and pharmacology needed to understand how organs function. Specific topics covered in Origins include receptor-mediated signaling and transport, muscle and neurophysiology, autonomic neuroanatomy, cancer biology, pharmacology basics, and the autonomic nervous system. The week-long autonomic nervous system portion of Origins is discussed in detail in the methods.

In the following semester, the Staying Alive course is an organ-system based course focusing on the physiology, pathology, and therapeutics within the cardiovascular, pulmonary and renal systems. In the beginning of the second week of Staying Alive, students explore cardiovascular autonomic physiology and are re-introduced to ANS pharmacology with a focus on a deeper application within the entire CV system.

In line with the Significant Learning principles ( Hurtubise and Roman, 2014), a first-year student in the class of 2022 developed an innovative worksheet for purpose of learning how ANS pharmacology manifests in the CV system. Discussed in detail in the methods, the innovative worksheet allows students to identify receptor specificity of cholinergic and adrenergic autonomic drugs, draw the heart rate (HR) and complete blood pressure profile (SBP, MAP, DBP) following administration, and predict the cardiovascular effects of two ANS drugs given simultaneously. The autonomic worksheet is a novel approach to practice ANS pharmacology and transfer their knowledge to a self-assessment problem set. Medical education is largely a worksheet desert, and this worksheet aims to fill this relative void. This study tests the hypothesis that the use of the ANS worksheet as a study tool improves outcomes of ANS questions on exams and self-assessments as well as boosts self-confidence. The class of 2023 was given the ANS worksheet in Origins and followed for the first year to survey their use, self-confidence improvement, and reported usefulness of the worksheet compared to the class of 2022, whom did not have access to the worksheet (control group). To our knowledge, this is the first worksheet of its kind to allow practice of autonomic cardiovascular pharmacology.

## Methods

### Autonomic Pharmacology Curriculum

The autonomic pharmacology portion of the curriculum is one week-long in the first semester Origins course and one week-long in the second semester Staying Alive course. Each week consists of multiple Peer Instructions (PIs), a simulation lab, and other sessions. The objectives of these learning events are:

• Compare and contrast the anatomy of the sympathetic and parasympathetic nervous system
• Identify the targets of the sympathetic and parasympathetic nervous system and explain how they respond to sympathetic and parasympathetic activation
• Compare and contrast the mechanisms of synaptic transmission in sympathetic and parasympathetic nervous system, from central origin to target, including receptors and intracellular signaling
• Categorize cholinergic and adrenergic autonomic medications by pharmacological class
• Explain how selective cholinergic and adrenergic pharmacological agents can manipulate the behavior of tissues, especially the cardiovascular system, innervated by the autonomic nervous system
• Predict side effects of cholinergic and adrenergic autonomic medications based on their mechanism of action

Student preparation for the three ANS Pharmacology days consisted of an ANS introductory video lecture, ANS anatomy video lectures, three peer instructions, and relevant readings from two physiology textbooks and a pharmacology textbook ( Costanzo, 2018; Koeppen and Stanton, 2018; Katzung, 2018; Trout, Borges and Koles, 2014; Dean Parmelee *et al.*, 2020). At the end of the initial ANS week, students participated in a three-hour simulation lab which provides real-life context to learning autonomic pharmacology and further solidify learning. During Origins course, two voluntary 5-question self-assessments (Assessment 1 & Assessment 2) were available online, spaced 72 hours apart, for students to evaluate their understanding of ANS pharmacology. During the Staying Alive course 11 weeks later, a voluntary 10-question self-assessment (Assessment 3) was available, consisting of the same re-arranged questions from Origins. A multiple-choice exam was administered at the conclusion of each week to assess application of neurophysiology, the ANS, and ANS pharmacology.

### Application of the Worksheet

**Figure 1.**
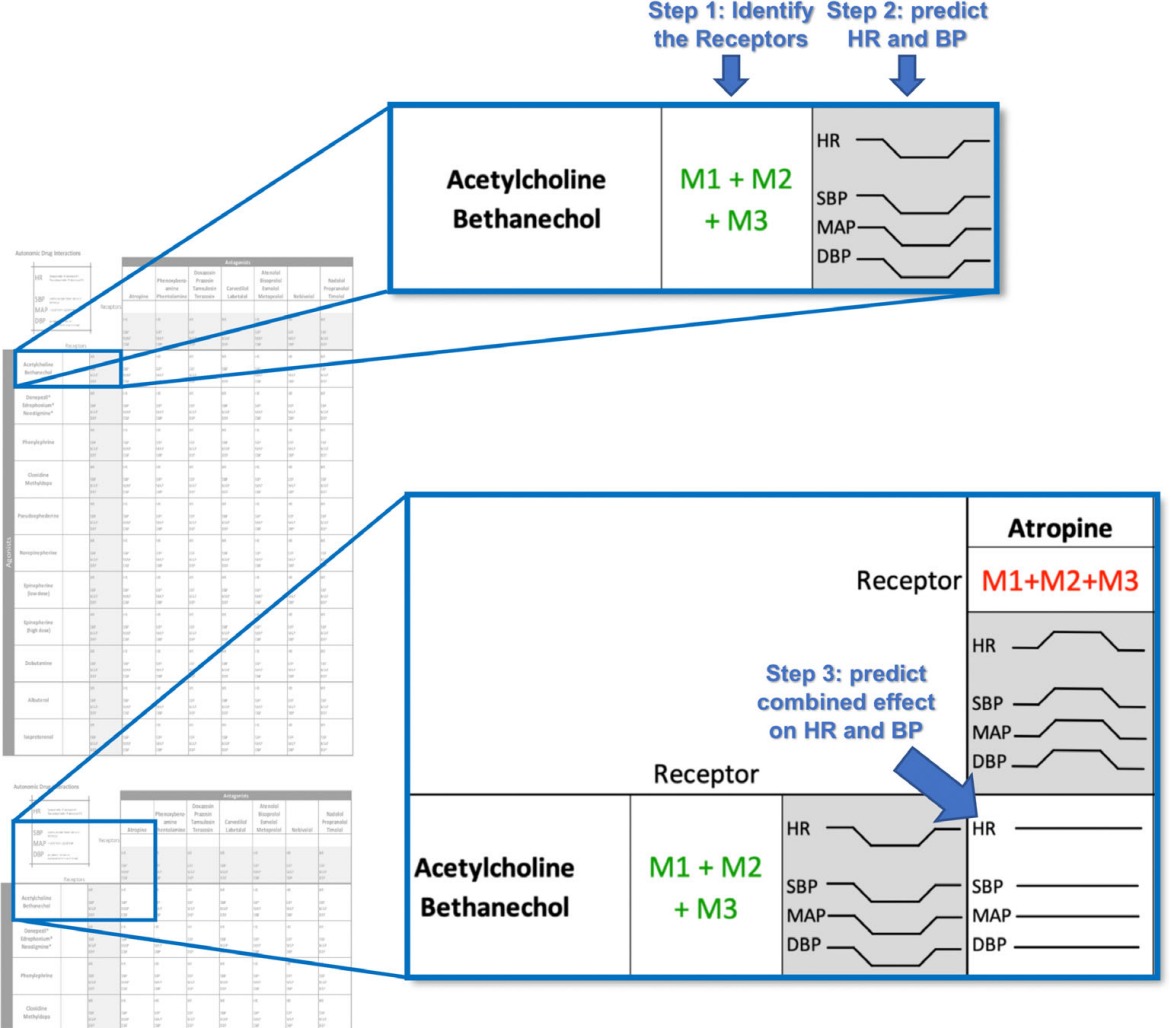
How to use the ANS worksheet

To supplement the curriculum, the ANS worksheet (Supplementary File 1) as well as the instructions (Supplementary File 2) were provided to the class of 2023 in PDF format with the intent for students to print out or use on a tablet and to work either individually or in groups. Further oral instructions and examples were provided in class. As illustrated in Figure 1, students initially draw the change in heart rate, systolic blood pressure, diastolic blood pressure, and mean arterial pressure (MAP) as a result of an intravenous administration of adrenergic or cholinergic drugs. Next, students use their knowledge of autonomic receptors and physiology to draw the effect of an autonomic agonist and antagonist administered simultaneously. This is designed to simulate a wide variety of NBME-style vignettes dealing with cardiovascular effects from adrenergic or cholinergic drugs given at the same time. An answer key was not provided to ensure students actively used available resources to fill out the worksheet, in lieu of copying from the key, and since many of the drug-drug interactions do not have a single agreed-upon answer (see limitations in discussion). Students had five days to use the worksheet as a study supplement before the Origins exam and were reintroduced to the worksheet before the Staying Alive exam. A non-mandatory review session in Origins was scheduled before the exam with purpose of teamwork on the worksheet.

Students identify drug-specific receptors and predict systemic effects by plotting drug effect vs time. For a full list of instructions, refer to the attached document.

### Surveying Usage, Usefulness, and Self-Confidence Improvement

Twice during the Origins ANS week and once during the Staying Alive ANS week, a voluntary survey was given at the end of each self-assessment (Supplementary File 3). The surveys asked students in multiple choice format to rate how much time they spent on the ANS worksheet, how useful they found it, and their reported confidence answering ANS pharmacology questions. Students were excluded from the study if they failed to complete all questions on all surveys and self-assessments.

### Assessing Outcomes on Exams

The Origins multiple choice exam consisted of 50 questions, of which 14 questions were specific to ANS pharmacology and relevant to the study. The Staying Alive multiple-choice exam also consisted of 50 multiple choice questions, 4 of which were specific to ANS pharmacology and relevant to the study. Although in randomized question order, the Origins and Staying Alive exams were identical between the class of 2022 and 2023.

## Results

### Survey and Self-Assessments

**Figure 2.**
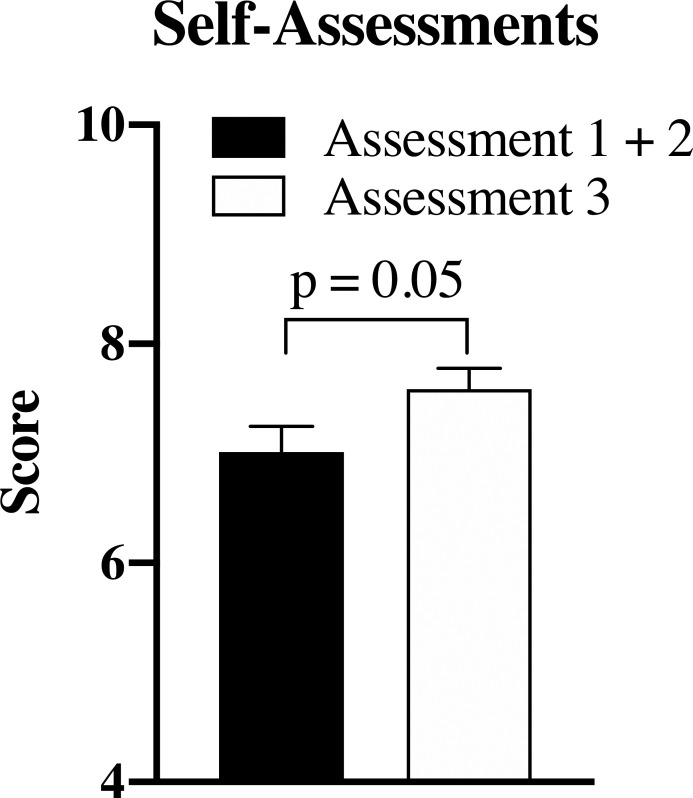
Scores for autonomic pharmacology self-assessments administered in Origins and Staying Alive

Student progress over time was monitored with three multiple-choice self-assessments. Assessments 1 and 2 consisted of 5 questions each and were released 72 hours apart in Origins. Assessment 3 was released in Staying Alive and, for valid comparison purposes, consists of the same 10 questions in randomized order from Assessments 1 and 2. In Figure 2, mean student scores were compared between Staying Alive and Origins, and a statistically significant mean score increase from 7.0 to 7.6 is noted (p = 0.05). Figure 2 reflects 68 students completing Assessments 1 + 2, and 75 students completing Assessment 3.

All participants stated use of the ANS Worksheet for at least 0.5 hour. The scores from the 5-question Assessments 1 and 2 in Origins were added together and compared to the 10-question Assessment 3 in Staying Alive. Questions are identical, and a significant increase in score is noted over time (p = 0.05). Data expressed as mean ± SEM.

**Figure 3.**
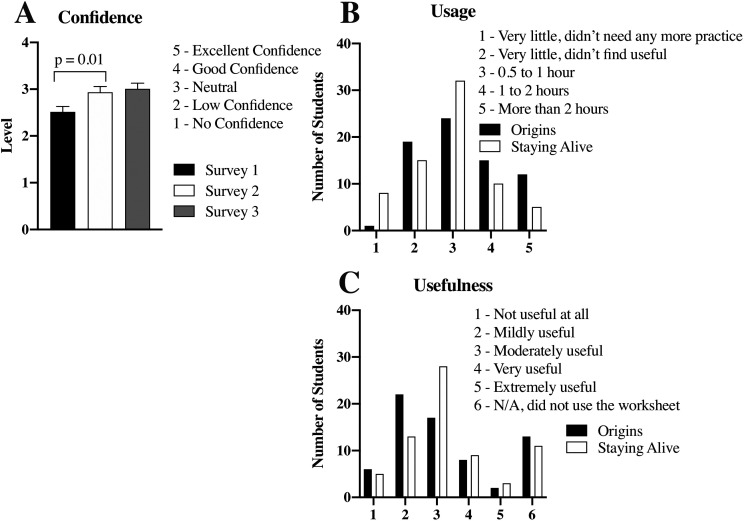
Self-reported confidence, usage, and usefulness of the ANS worksheet

Figure 3 displays student confidence, usage, and usefulness of the ANS worksheet. Self-confidence in answering ANS pharmacology questions was monitored in Assessments 1, 2, and 3. A significant increase in self-confidence was reported among worksheet users between Assessments 1 and 2, reflecting a rapid boost in confidence within 72 hours of exposure to ANS pharmacology questions. The increase in confidence between Assessments 2 and 3 is statistically not significant, reflecting similar confidence levels in the following semester’s Staying Alive course.

(A), student confidence pertaining to answering ANS pharmacology questions was surveyed at three timepoints throughout the first year of medical school. A significant increase in student confidence is noted between Surveys 1 and 2 (p = 0.01). Surveys 1 and 2 were administered one week apart in Origins, while Survey 3 was administered in Staying Alive. Data expressed as mean ± SEM.(B) and (C), frequency distribution of student-reported times spent using the worksheet and rated usefulness of the worksheet, respectively. Students were surveyed once in Origins and once in Staying Alive.

Usage and reported usefulness of the ANS worksheet as a study tool was monitored once in Origins (Assessment 2) and once in Staying Alive (Assessment 3). Figure 3B- C shows frequency distributions of 75 survey participants reporting usage and usefulness of the worksheet as a study tool. The most reported use of the worksheet fell between 0.5 and 1 hour in Origins and Staying Alive; however, more students in Staying Alive used the worksheet for longer than one hour compared to Origins. The average student usefulness score was “moderately useful” in Origins and Staying Alive; however, more students in Staying Alive rated the worksheet “very useful” and “extremely useful” than did in Origins.

### Exams

**Figure 4.**
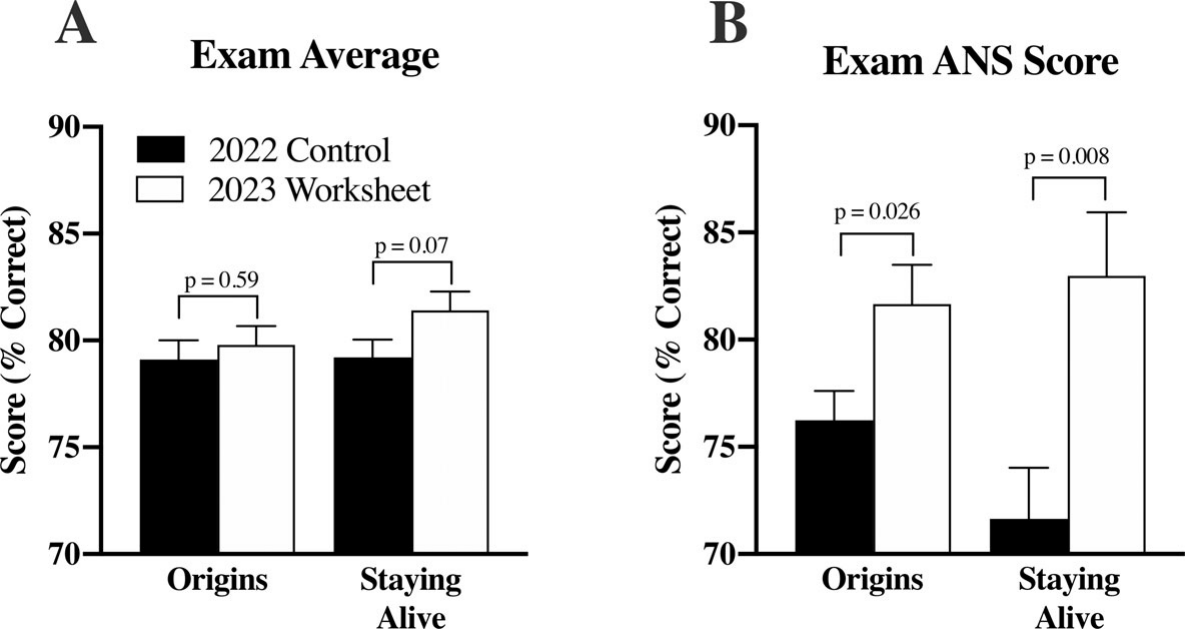
Scores for 50-question exams without and with use of the ANS pharmacology worksheet as a study tool

A 50-question multiple choice exam was administered to conclude each ANS week in Origins and Staying Alive. Students in the class of 2022 and 2023 were administered randomized, but identical exam questions, allowing outcome analysis of the worksheet as an effective study tool in answering ANS pharmacology questions. These exams included questions pertinent and non-pertinent to ANS pharmacology. Figure 4A displays overall exam scores in Origins and Staying Alive and shows no significant changes in exam scores between the class of 2022 and 2023 (p > 0.05). The Origins exam had 14 questions pertaining to ANS CV pharmacology and the Staying Alive exam had 4. These specific questions serve as outcome measures. Figure 4B compares the percent correct of ANS CV pharm questions between the class of 2023 (worksheet group) and the class of 2022 (no worksheet, control). For the Origins exam, students in the class of 2023 significantly outperformed their 2022 counterparts by 5.4 points (p < 0.05). For the Staying Alive exam, students in the class of 2023 with access to the worksheet scored 11.3 points higher than the class of 2022 did (p < 0.01).

(A), No statistically significant difference in overall exam scores are noted without (2022) and with (2023) the use of the worksheet. (B), however, highlights the significant increase in score of ANS pharmacology questions pulled from these exams between the class of 2022 and 2023. Fourteen questions from the Origins exam and four questions from the Staying Alive exam are ANS pharmacology questions, while the remaining questions focus on other topics out of scope from the study. Data expressed as mean ± SEM.

### Statistical Methods

Survey and self-assessment participants were volunteers, and only the class of 2023 had access to the worksheet. In Figures 2, 3A, and 4, a two-tail t-test was performed for analysis in GraphPad Prism 8.

## Discussion

During the first year of medical school, students using the ANS pharmacology worksheet as a study tool saw an increase in self-assessment and exam outcomes compared to those who did not use the worksheet. Student outcomes were backed up by promising student usage, self-confidence, and usefulness of the worksheet. To our knowledge, this worksheet is the first of its kind to allow students to actively learn autonomic pharmacology by predicting the cardiovascular effects of ANS drugs when given as monotherapy and simultaneously as dual therapy. Predicting the effects of dual therapy allows users to hone their understanding of competing agonism and antagonism of ANS receptors. The results show a promising adjunct to the Wright State University BSOM autonomic pharmacology curriculum.

Across many US medical schools, intense effort has been made to determine the effectiveness of active learning, compared to the traditional lecture-based system, and its place in medical education. Deslauriers *et al*. notes students who are in an active learning environment learn more effectively but perceive they learn less than those in a passive learning environment( Deslauriers *et al.*, 2019). Active learning allows students to uncover their own misconceptions and construct their own correct explanations. One reason students dislike active learning, however, is their perceived disfluency associated with the increased cognitive effort put forth, which may actually have a negative impact on their actual learning. In order to make active learning effective, Deslauriers *et al*. recommends instructors highlight the proven effectiveness of active learning so that students accept that it leads to deeper learning and acknowledge that it sometimes feels counterintuitive. In our study, we demonstrated the effectiveness of active learning from the worksheet by measuring student outcome on exams and self-assessments. Simultaneously, we were able to capture their perception of this active learning strategy by surveying student confidence and perceived usefulness of the worksheet. Student appreciation of active learning and high level of confidence are essential for active learning to be effective. Student users of the worksheet showed an increasing confidence level over time as well as moderate appreciation or usefulness of the worksheet, attesting to the successful implementation of the worksheet into the BSOM curriculum.

Nationwide efforts have been made, especially over the past decade, to enhance undergraduate medical education towards creating meaningful active learning experiences that foster retention and a passion for life-long learning. Incorporation of Peer Instructions and Team-Based Learning activities into the BSOM Foundations period are two proven effective approaches towards meaningful learning ( Trout, Borges and Koles, 2014; Parmelee *et al.*, 2020; Parmelee and Hudes, 2012). These approaches incorporate learning strategies such as immediate feedback, retrieval-based learning, self-explanation, practice testing, and elaborate peer interrogation. The student developed ANS pharmacology worksheet was designed with these same learning strategies in mind. With the worksheet, students are able to practice with the listed 32 autonomic drugs and the 77 drug interaction scenarios covered. In peer groups, students have the ability to aid each other in solving complex ANS drug interaction scenarios, compare answers, and provide feedback. Thinking beyond the immediate effects of a drug is a critical step in understanding pharmacology. A major advantage of this worksheet is it allows users to not only identify the drug-specific receptors, but also to critically think through the cellular effects, identify patterns, and integrate their understanding with the whole cardiovascular ANS system.

This worksheet - designed by students, for students - successfully serves as an adjunct study tool in the cardiovascular autonomic pharmacology curriculum by providing a way to practice, build confidence, and develop a deeper understanding into the drug-receptor-system relationship.

### Limitations

This worksheet is intended to be solved with the understanding that each HR and BP effect is perceived in an idealized experimental environment. Many of these drug combinations are not used in clinical medicine but are included in effort to further student understanding of the drug-receptor-system relationship. Many of these drug interaction effects have not been experimentally proven and may not have a clear answer; however, the intent here is to use the simple, core principles of autonomic pharmacology to predict the effects. The extent to which each drug changes HR and BP parameters is not as important here. For example, some students may predict a reflex bradycardia when pseudoephedrine and atropine are administered simultaneously, while others do not. The study may also be limited due to the intended voluntary participation in the worksheet and survey.

## Take Home Messages

By the end of this session, students should be able to:

• Utilize the worksheet as a novel method to apply their knowledge of autonomic receptor molecular biology with cardiovascular physiologic properties.
• Anticipate the effects of individual autonomic drugs based on their affinity for various autonomic receptors.
• Create opportunities for self-assessment of how autonomic drugs, when administered simultaneously, have agonistic and antagonistic properties on cardiovascular autonomic receptors and apply these drug-drug interactions to a physiological level.

## Notes On Contributors

**Christopher Cooley** is a third year medical student at the Wright State University Boonshoft School of Medicine. ORCID ID: https://orcid.org/0000-0002-3733-208X

**Dr. Mary Jo Trout** is an Assistant Professor in the Departments of Geriatrics and Pharmacology & Toxicology and Director of Therapeutics at the Wright State University Boonshoft School of Medicine. ORCID ID: https://orcid.org/0000-0002-6194-8095

## Declarations

The author has declared that there are no conflicts of interest.

## Ethics Statement

This study falls under IRB exemption #SC0653 for medical education research at the Wright State University IRB office. The research was conducted in accordance with the Declaration of Helsinki and the MedEdPublish Protection of Research Participants policy.

## External Funding

This article has not had any External Funding
