# Supplementary material for: Teaching Autonomic Cardiovascular Pharmacology to Medical Students: An Innovative Worksheet Approach
Source: MedEdPublish (2016). 2021 Mar 8;10:65. [Version 1] doi: 10.15694/mep.2021.000065.1 (PMC10939634; doi:10.15694/mep.2021.000065.1)
Supplement: Peer review history

# Reviewer report: Reviewer response for version 1

**DOI:** 10.21956/mep.18872.r26927

**Published:** 2021-10-05

**Authors:** Stijntje Dijk

**Affiliations:** 1 Erasmus Medical Center

This review has been migrated. The reviewer awarded 4 stars out of 5 Many congratulations to the author for their work to provide this paper as well as the worksheet as a thought-out, student-centered, open-access available resource for both staff and students. At times, medical education journal articles will mostly contain general methodology papers, whereas this paper provides a welcome, topic and skill-specific concrete example of learning material that would be implementable in many setting.My note on this paper is that I am missing a critical reflection on the research study aspect in the discussion section. Please note that, regardless of the bias or scientific merit of the findings, I do think that if even one student believes they benefit from the use of the tool, which would be at no cost/effort to implement, the publication of this sheet is a success. From a more critical appraisal of the design of the study, there are various potential sources of bias or other distorting factors. Voluntary surveys may lead students who did worse/better to be more likely to fill or submit them, and students doing well with the worksheet may be more willing to demonstrate the merit of it and hence fill out the survey. There may be a spillover of students studying together with and without the tool. Students are not randomized to using the tool or not, meaning that it is possible that more engaged/eager students or students who would have also obtained better results without the worksheet may have been more likely to use it. A consideration for future cohorts may be to think about what the appropriate counterfactual would be, and whether you would like to look at your results based on the actual usage of the worksheet, or availability of the worksheet. The current paper could benefit from an addition to the discussion that would provide a critical reflection of the research aspect of the tool.Congratulations to the authors on this paper, and specifically the medical student author/designer of the worksheet.

Reviewer Expertise:

NA

No decision status is available

# Reviewer report: Reviewer response for version 1

**DOI:** 10.21956/mep.18872.r26926

**Published:** 2021-03-14

**Authors:** Linda Costanzo

**Affiliations:** 1 Virginia Commonwealth University

This review has been migrated. The reviewer awarded 5 stars out of 5 It was a treat to review this novel project devised by a third year medical student at Wright State University Boonshoft School of Medicine! The premise is, that by incorporating active worksheets in phased learning of the autonomic nervous system, students’ confidence and accuracy in applying these principles to the cardiovascular system will be enhanced. More than one-half of the medical class of 2023 (M’23) voluntarily used a supplementary worksheet tool that asked them to predict changes in heart rate and blood pressure in response to autonomic drugs given singly or in combination. Since the worksheet required that students draw the predicted changes, they could not rely on simple recall or cues from multiple choice questions. Active learning in action! The study controls are appropriate, as the authors compared the performance on autonomic pharmacology exam questions of participating and non-participating members of the Class of 2023. In addition, exam performance of participating students was compared with that of the entire class of 2022 who did not have access to the tool. Performance on autonomic questions was significantly enhanced in both the first year “Origins” course as well as the second year “Staying Alive” course. It is interesting that surveys of participating students showed a much higher confidence level in applying these complex, higher-order concepts. In my opinion, the strength of this study is, in the authors’ words, that it was “designed by students, for students.” As the authors point out, active learning methods are often met with student-skepticism regarding cost (time) vs benefit (exam outcome). This study addresses that potential skepticism head-on by demonstrating both perceived value by students and improved exam results. The target audience is medical educators and medical students interested in devising active methods for learning and applying difficult concepts. It certainly inspired me to ask: what other areas of applied physiology and pharmacology might lend themselves to an active worksheet? Hm…….

Reviewer Expertise:

NA

No decision status is available
